# Retraction of Nursing scientific publications

**DOI:** 10.1590/1518-8345.0000.3921

**Published:** 2023-04-17

**Authors:** Maria Lucia do Carmo Cruz Robazzi, Sandra Valenzuela Suazo

**Affiliations:** 1 Universidade de São Paulo, Escola de Enfermagem de Ribeirão Preto, Centro Colaborador de la OPS/OMS para el Desarrollo de la Investigación en Enfermería, Ribeirão Preto, SP, Brasil; 2 Universidad de Concepción. Facultad de Enfermería, Departamento del Adulto y Adulto Mayor, Concepción, Chile

**Figure:**
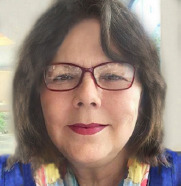


**Figure:**
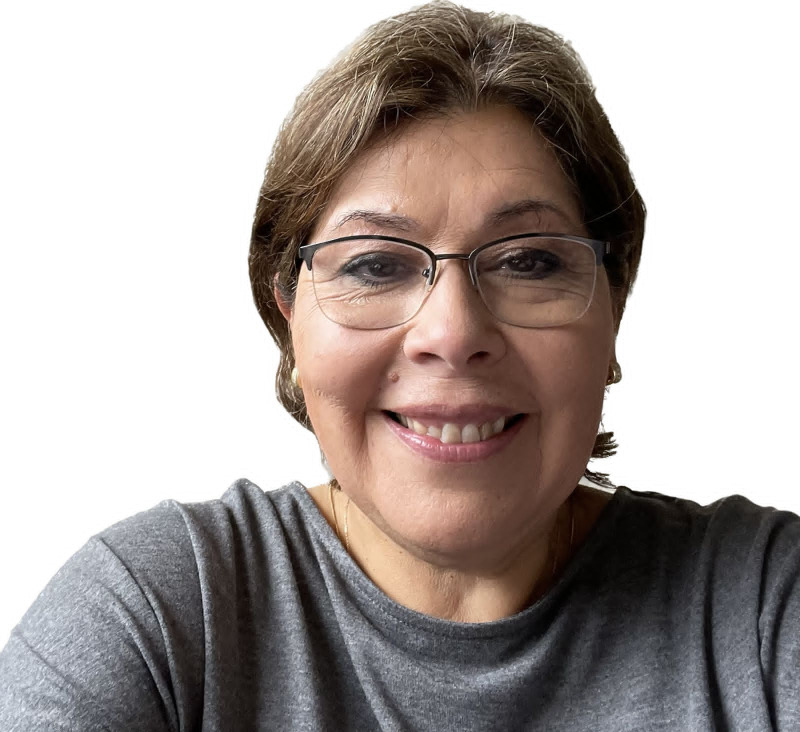


The word “retraction” derives from Latin “ *retracto*- *are*”: undertake again, resume, correct, withdraw; it means taking back what was said, refuting what was said, unsaying, apologizing ^( 1)^ , acknowledging a mistake ^( 2)^ . In many situations, there is a need to retract something, even in the scope of science, involving publications, for example. Retracting published scientific texts is not a new fact; it has been a reality in the academic/scientific environment for years. 

A retracted publication can be an article or book retracted in its entirety or in full by one or more authors or by an authorized representative. The author identifies a previously published citation and retracts it by means of a formal publication, authored either by himself/herself, the editor, or another authorized agent ^( 3)^ . In turn, retraction of a publication is a statement published by one or more authors of an article or book, withdrawing or disavowing their participation in performing the research or recording the results of their study in writing ^( 4)^ . 

Publication retractions occur when the scientific findings are no longer considered reliable due to misconduct or scientific error, plagiarism of previously published studies, or violation of ethical guidelines ^( 5)^ . When unethical behaviors such as data fabrication, lack of the participants’ consent, methodological problems, cloned data and plagiarism are suspected and/or confirmed, among other situations, what was published should be retracted. But it is also the case when the article is misleading and lacks a theoretical framework to support the assertions made and when there is bias, conflict of interest, and tendentious data ^( 6)^ . 

Most of the times, peer reviews requested by the editors contribute to clarity and quality of an article submitted to a journal, oftentimes detecting errors and incorrect interpretations ^( 7)^ . However, in some assessments there is feedback with weak opinions that hardly help to improve the quality of the article under evaluation. 

Therefore, it is noticed that this practice is becoming increasingly more common, due to the pressure to meet publication deadlines and to editorial failure previously identify problems with the submitted, approved and published texts, including cases of plagiarism and research data fabrication ^( 8)^ . 

In addition to the aforementioned, one of the possible problems in relation to retracted texts is the possibility of them being cited by other authors, before the due retraction. For example, it is important for Nursing researchers to request that their graduate advisees evaluate the references of their dissertations/theses to identify possible retraction cases.

A researcher examined the extent of the problem in the Nursing field, identifying 23 systematic reviews that included retracted studies. Considering a clinical discipline such as Nursing, professionals strongly rely on systematic reviews to inform their care and treatment decisions; thus, if a retracted study is included, integrity of the review should be questioned ^( 9)^ . 

Retracted articles are found in databases that are traditionally used by researchers in Nursing, such as *Scopus, National Library of Medicine* and *Embase*, among others. 

In the scope of national Nursing journals researched on the web, some retractions by both authors and editors were identified: acknowledgment of verbatim copy of a scientific article by other author, without due credit; identification of an article that had already been published in the same journal; failure to attribute authorship to all individuals involved in the reported and published experience; duplicate submission of a text published in a national journal and its subsequent publication in an international journal; total retraction and removal of an article from circulation due to noncompliance with the good practices for responsible use of the information, among other problems.

The search on the Retraction Watch website ( https://retractionwatch.com/), a database that catalogs study corrections made in several journals worldwide, revealed some situations that required retraction from Nursing researchers, as shown below. 

A study published in 2008 referring to 12 years of research on Nursing education was contested/criticized more than 10 years after its publication and citation by several other authors and is still under contestation and awaiting responses. Retractions/Erratum notices referring to Nursing researcher that published articles as first author, most of them duplicates, use of data without ethical approval, parts of the article that had already been published by the author himself and by his research group, as well as several texts containing these retractions, had already been cited by other authors.

The clinical value of honey on venous ulcers was tested in a study that had to be retracted by the journal that published it, after an investigation uncovered “errors in data analysis”, although it had already been cited 38 times; more recently, the same authors submitted another article, which also required retraction.

An article on the growth hormone published in 2013 that analyzed inefficiency in the use of devices for administering this hormone was retracted upon request of the very pharmaceutical company where the authors worked.

A controlled randomized study on Pediatric Surgery was retracted in 2021, after a reader raised doubts about the methodology described in it. Based on the observations made by the reader, several other problems were found in the article, including lack of clarity on sample size calculation, generating mistrust in the interpretation of this study and in its explicit recommendations for the practice. Problems of this nature might and probably should, have been detected during the peer review process, which was not the case.

An author had his article from 2019 retracted in a Nursing journal after the editors learned that the researcher had used unauthorized data obtained from the university where he had graduated as a PhD. However, the article had already been cited seven times before being retracted.

The topic is valuable and worthy of other reflections that may show several of its aspects. The scenario points to weaknesses that still exist in editorial systems, favoring the publication of articles with ethical infringements such as data collection frauds, omissions, plagiarisms, real and/or ghost authors and conflicts of interest, which subsequently had to be retracted, in all knowledge areas, including Nursing. The problems affect authors, reviewers, editors and readers, who eventually detect the problems and inform the authors and/or the journal, thus weakening the Nursing science.

Retractions are self-correction and correction mechanisms inherent to science ^( 10)^ , which is an essential element and should be built and supported on its own scientific process and progress. 

## References

[1] Dicionário Priberam (2021). Retratação.

[2] Infopédia (2021). Retratação.

[3] Descritores em Ciências da Saúde (2023). Publicação Retratada.

[4] Descritores em Ciências da Saúde (2023). Retratação de Publicação.

[5] Fang FC, Casadevall A (2011). Retracted science and the retraction index. Infect Immun.

[6] Pichinin L (2020). Retrospectiva de retratações científicas.

[7] Cintas P (2016). Peer review: from recognition to improved practices. FEMS Microbiol Lett.

[8] Pires GL, Poffo BN (2017). A avaliação da pós-graduação em educação física e suas implicações para os periódicos da área: “publicar ou perecer” vale também para os editores. Rev Educ Física.

[9] McCook A (2018). A concerning – largely unrecognised – threat to patient safety. Nursing reviews cite retracted trials’.

[10] Watanabe EH (2014). A não linearidade entre a reação de quem copia e de quem é copiado. Estudos Avançados.

